# Understanding how digital training enhances healthcare worker perceptions of HIV index case testing: a qualitative explanatory analysis

**DOI:** 10.21203/rs.3.rs-8853616/v1

**Published:** 2026-03-22

**Authors:** Tapiwa A. Tembo, Milenka Jean-Baptiste, Tiwonge Mbeya-Munkhondya, Mtisunge Mphande, Mike J. Chitani, Angella Mkandawire, Caroline Kumbuyo, Katherine R. Simon, Saeed Ahmed, Vivian F. Go, Linda-Gail Bekker, Nora E. Rosenberg

**Affiliations:** Baylor College of Medicine Children’s Foundation; University of North Carolina at Chapel Hill; Kamuzu University of Health Sciences; Baylor College of Medicine Children’s Foundation; Baylor College of Medicine Children’s Foundation; Baylor College of Medicine Children’s Foundation; Baylor College of Medicine Children’s Foundation; Baylor College of Medicine Children’s Foundation; Baylor College of Medicine Children’s Foundation; University of North Carolina at Chapel Hill; University of Cape Town; University of North Carolina at Chapel Hill

**Keywords:** Index case testing, partner notification, lay health workers, digital training, explanatory analysis, LMIC, videos, checklists

## Abstract

**Background:**

HIV index case testing involves offering HIV testing services to the sexual partners and children of people living with HIV. In Malawi, index case testing implementation has been suboptimal due to inadequately trained health care workers (HCW). In a cluster-randomized trial, we tested the impact of a digital training approach (Enhanced strategy) to improve HCW capacity to conduct HIV index case testing services. We conducted a qualitative explanatory analysis to understand the impacts of the training on health workers’ perceptions of delivering the intervention.

**Methods:**

In a cluster randomized trial, 33 clusters (health facilities) were randomized 1:2 to the Enhanced and Standard arms. HCWs in both arms received brief centralized in-person training on index case testing. HCWs in the Enhanced arm also received decentralized digital training through 20 synchronous and asynchronous sessions, which provided knowledge on protocols and principles of index case testing, modelled counseling scenarios, practice counseling, and individual feedback. We conducted 26 in-depth interviews with HCWs from 14 clusters (n = 6 Enhanced, n = 8 Standard) in the weeks after the Enhanced training. Interviews focused on HCW perceptions of their work. Interview transcripts in the weeks after training were analyzed thematically using an inductive approach. We compared responses of HCWs from Enhanced and Standard arms.

**Results:**

Overall, HCW in the Enhanced arm perceived greater ease counseling clients after the training. This was attributed to five facilitators:1) HCWs perceived the checklists as helping them operationalize ICT, 2) Video vignettes facilitated new counseling skills, 3) HCWs expressed greater comfort and confidence handling a range of clients, 4) HCWs perceived their counselling skills were more impactful, and 5) HCWs expressed greater confidence identifying and solving facility-level challenges. HCWs from the Standard arm requested additional tools, and referred to more experienced providers, reflecting less confidence in index case testing implementation.

**Conclusions:**

Digital training was viewed as a mechanism for enhancing knowledge, counselling skills, and comfort and confidence to address facility-level challenges. Digital training with video vignettes, and checklists to guide practice and feedback sessions are key strategies for HCWs capacity-building in low-resource settings.

**Trial Registration Number:**

NCT05343390

## Background

Human Immunodeficiency Virus (HIV) remains a public health concern, with approximately 41 million people living with HIV (PLHIV) globally, including 13% who do not know their status [1, 2]. The Joint United Nations Program on HIV/AIDS (UNAIDS) established operational goals for national HIV programs to diagnose ≥ 95% of those living with HIV, provide HIV treatment to ≥ 95% of those diagnosed, and achieve viral load suppression in ≥ 95% of those receiving treatment, known as the UNAIDS 95–95-95 targets [3]. However, in Malawi in 2021, 88.3% of PLHIV were aware of their status, lagging behind the first of the UNAIDS targets. HIV diagnosis is the first step to offering prevention and treatment services for PLHIV. Index case testing, also known as partner notification, is an effective case-finding strategy where health care workers (HCWs) interview PLHIV about their sexual partners and biological children and support these contacts with accessing HIV testing services [4–6].

HCWs capacity to deliver index case testing is one key consideration for optimal implementation. However, our formative work conducted in 2019 revealed that HCWs had limited capacity to deliver index case testing [7]. Notably, HCWs reported feeling uncertain discussing sexual partnerships and explaining index case testing procedures in a client-centered and culturally appropriate manner, a task that requires skills and adequate training [8]. HCW supervisors suggested additional teaching, modeling, practicing, and receiving feedback would enhance HCWs’ skills [7].

Standard capacity-building for HCWs is centralized, synchronous, and in-person training. However, this approach is costly, logistically challenging and prone to variability in quality due to different trainers’ teaching ability [9]. A digital training using “blended learning” (combination of digital and in-person training) could overcome these challenges and enhance capacity in HCW [10–12]. Digital training can be asynchronous, decentralized, and delivered electrically (via tablet) reducing the need for travel, lodging and facilitators. However, there is limited evidence evaluating the impact of digital training for HCWs on implementation and clinical outcomes in low- and middle-income countries—especially surrounding counseling skills.

To address this gap, we developed a digital training based on social cognitive theory [13] and the theory of expertise [14], emphasizing the roles of teaching, modelling, practice, feedback, and quality improvement in acquiring knowledge and behavioral skills. The training was pilot tested in 2019 and showed promising results [15]. Thereafter, we conducted a cluster randomized controlled trial called PRACTICE (Package of Resources for Assisted Contact Tracing: Implementation, Cost, Effectiveness), which aimed to examine the implementation and effectiveness of a digital training strategy compared to standard of care for index case testing in Malawi [16]. The PRACTICE study primary analyses demonstrated that the digital training improved HCW knowledge, skills, counseling behavior, and clinical outcomes, including rates of contact client elicitation, testing, and diagnosis [17, 18].

The purpose of this analysis is to understand the mechanisms and processes by which digital training improved knowledge, skills, behavior, and clinical outcomes from the perspective of HCWs delivering index case testing. Therefore, we conducted a qualitative explanatory analysis to explore HCWs’ perceptions of the mechanisms that contributed to the observed outcomes in index case testing and changes in HCWs’ experience after the digital training.

## Methods

### Study setting

The PRACTICE study was conducted in health facilities in Balaka and Machinga districts in Malawi. The facilities provide free HIV testing and treatment services, including HIV index case testing. Malawi has a low rate of certified HCWs (< 5 clinicians per 100,000 people), resulting in task-shifting of HIV testing services to lay HCWs with minimal formal training [19]. The majority of lay HCWs at the study facilities were employed by Tingathe Program, a United States President’s Emergency Plan for AIDS Relief (PEPFAR)-supported program supporting the Malawi Ministry of Health to deliver HIV care and treatment services [20]. As part of index case testing, HCWs were expected to interview PLHIV, elicit contact clients’, provide referral options, and collect contact information for each client. Contact clients were traced either via phone or in the community and offered HIV testing services or linked to treatment.

### Parent trial

The qualitative sub-study was part of the PRACTICE study, a two-arm cluster-randomized controlled trial that evaluated the impact of a digital training on HCW capacity for index case testing [16]. In the PRACTICE study, 33 clusters (health facilities) were stratified into six randomization strata based on district, facility level, and performance. Within each stratum, the clusters were randomized in a 1:2 ratio to Enhanced arm (11 clusters) vs Standard arm (22 clusters) by an independent statistician using an algorithm coded in SAS version 9.4 (Cary, NC). Randomization assignments were revealed to clusters and study staff after HCW enrollment and immediately before enhanced arm training activities. Due to the nature of the implementation strategies, study staff, participants, and investigators were not blinded to the study arm. The PRACTICE study adheres to CONSORT guidelines.

The Standard arm was trained on key aspects of the ICT program. The Enhanced arm received the standard training plus a digital training implementation strategy. There were no changes to trial design and outcomes during implementation.

### Enhanced arm

**Figure 1:**
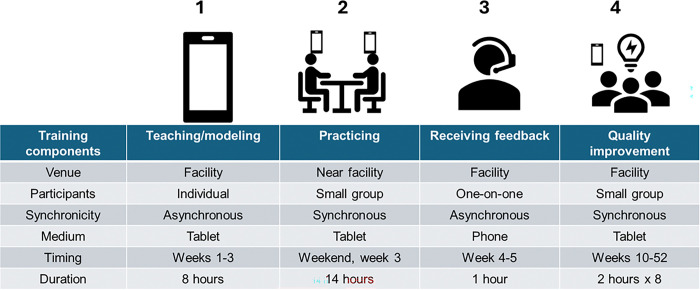
The digital training implementation strategy

The digital training consisted of teaching/modelling, practicing, feedback, and quality improvement and is described in detail elsewhere [16]. Briefly, the teaching and modelling consisted of eight hours of watching videos of an expert counselor model different counseling scenarios with fictional index and contact clients. The practicing was 14 hours of tablet-guided practice sessions using 15-item checklists —one for index clients and the other for contact clients. The checklists consisted of introduction, building rapport, confidentiality, explaining the purpose of index case testing (ICT), and discussing testing approaches. The feedback session reinforced the lessons through a mock counseling session. The quality improvement sessions involved orienting the HCWs on identifying barriers to ICT and discussing workable solutions as a team. The training development was guided by the social cognitive theory (SCT) [13], which suggests that learning occurs in a social context with reciprocal interactions between personal factors, behavior, and environment. Observing index case testing video vignettes that model counseling could facilitate social learning, practicing internalize behavioral skills and improve self-efficacy, and receiving feedback reinforces these skills (Fig. 1).

### Nested qualitative research

**Figure 2:**
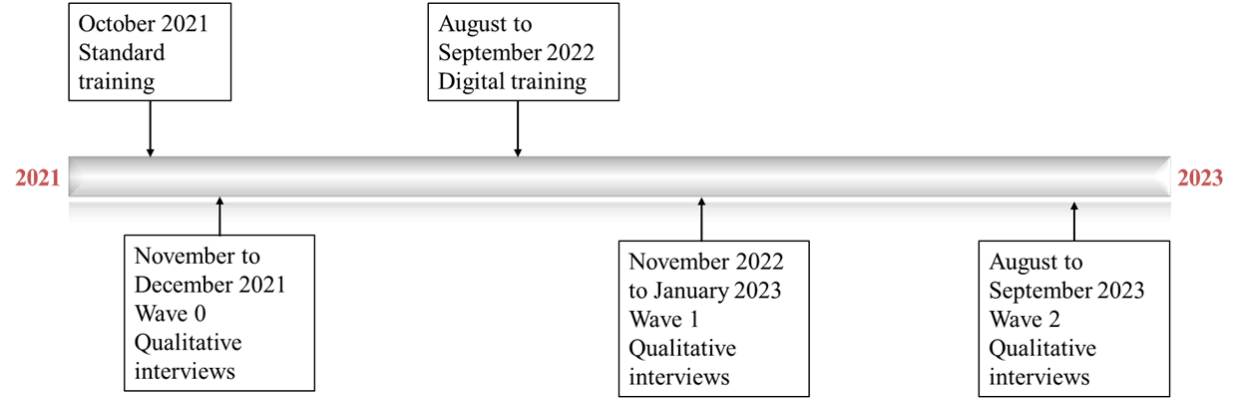
Timeline of the training and qualitative interviews

The qualitative research was conducted with HCWs from both arms to explore differences in HCW experiences implementing index case testing services over time. We purposively selected facilities from each stratum that were from both arms, both districts, and different levels of care. 26 health workers from 14 clusters (6 = Enhanced, 8 = Standard) were invited to participate. HCWs were ≥ 18 years old and involved in the index case testing program. Research staff recruited HCWs in November 2021. Each HCW participated in three in-depth interviews (IDI) at three different time-points: before training, in the weeks after training, and one year after training (Fig. 2). In this analysis, we used data from the weeks after the training (November 2022 to January 2023).

### Data collection procedures

Two trained Malawian qualitative researchers (TM and MM) who were independent of the implementation team conducted the in-depth interviews (IDIs) using a semi-structured interview guide developed for the study (Supplementary File 1). The interviews were conducted in Chichewa, the primary local language in Malawi. Both interviewers were female, had university-level education, and were fluent in Chichewa.

The interviews for this analysis focused on HCWs’ perceptions of the digital training implementation strategy (Enhanced arm only) and counseling index and contact clients (both arms). The interviews were conducted in a private and quiet setting. Most of the interviews were conducted via phone, and a few were conducted in person. IDIs were audio-recorded and lasted 60 to 90 minutes. Interviewers summarized the IDI data after the interviews.

The wave 1 interviews were done in two phases. In the first phase, interviewers asked about views of each of the digital training components: teaching through videos, practicing, receiving feedback, and quality improvements (Enhanced arm only). In the second phase, interviewers asked about the views on providing counseling to index and contact clients, probing on experiences interacting with different types of clients and perceived changes after the training. Social harms were assessed during the study implementation.

### Data management and analysis

IDI audio recordings from each wave were transcribed and translated directly from Chichewa into English by professional Malawian transcribers fluent in both Chichewa and English. The research team reviewed transcripts for completeness and correctness. Identifying information was removed.

Data were analyzed thematically [21]. The lead author (TAT) read the transcripts to become familiar with the data. The lead author then developed a codebook based on the transcripts and interview summaries with input from two other researchers (MJC and AMM). The codebook consisted of deductive codes drawing from the interview guide and inductive codes emerging from the data. One set of codes mapped to the Social Cognitive Theory. TAT coded the transcripts using NVivo version 19. During the coding process, the codes were periodically discussed with two other researchers (MJC and AMM) to identify emerging patterns. The coded texts and code reports were reviewed and organized in matrices in MS Excel to further identify patterns in the data. The identified themes were reviewed and discussed with additional team members (MJB and NER). The lead author selected illustrative quotes that best represented each theme, and comparisons were made between arms.

### Patient and Public Involvement

The digital training implementation package was developed in consultation with key Tingathe Program stakeholders and the Malawi Ministry of Health to ensure content is acceptable, informative, and engaging.

#### Ethical Approval

The study was reviewed and approved by the National Health Sciences Research Committee, the University of North Carolina Institutional Review Board, and the Baylor College of Medicine Institutional Review Board. Informed consent was obtained from all participating HCWs, and they were compensated for their participation in each interview. De-identified data and a data dictionary are publicly available on the UNC dataverse at https://doi.org/10.15139/S3/KEPG04.

## Results

### Participants characteristics

The qualitative sub-study was conducted in 5 clusters in Balaka and 9 clusters in Machinga. There were six clusters in the Enhanced arm and eight in the Standard arm. Ten clusters were rural and four semi-urban/urban. Nine were health centers, two were secondary-level hospitals, and three small dispensaries.

There were 26 participants: 15 from the Standard arm and 11 from the Enhanced arm. HCW median age was 30 years (IQR:25–34). Most were male (65%) and had completed secondary education (85%). Median years of job experience was 4 (IQR:3–5) (Table 1). There were no reported social harms.

### Findings

The analysis identified five themes and the results are presented below by theme: 1) HCWs perceived the checklists as helping them operationalize ICT, 2) Video vignettes facilitated new counseling skills, 3) HCWs expressed greater comfort and confidence handling a range of clients, 4) HCWs perceived their work was more impactful, and 5) HCWs expressed greater confidence identifying and solving facility-level challenges (Table 2).

#### Theme 1: HCWs perceived the checklists as helping them operationalize ICT

HCWs in the Enhanced arm described how the checklists were helpful as they provided the step-by-step guidance on counselling index and contact clients. They mentioned using the study checklist to walk the client through the counseling session step by step. HCWs expressed being able to know where the session should start and how to end it, which was previously not done systematically. They reported the checklist as the guide that made index case testing counseling easy. In the Standard Arm, HCWs requested guidelines for the counseling session to make the index case counseling easier and more effective. One participant mentioned, *“By having the guideline so that you can be able to follow.”* This was requested to support them when conducting the session.

The first part of the index client checklist consisted of introducing the session, explaining confidentiality, and seeking consent. This was followed by exploring the client’s need for index case testing. One HCW described, “*On the checklist, we build rapport with the participant so that she or he feels free. We assure the client of confidentiality and we seek consent from the client.”*

On the other hand, Standard arm HCWs explained that they first focus on emphasizing the importance of eliciting contacts. This was viewed as a way for the client to understand the purpose of index case testing and be willing to disclose their contacts. One HCW described, “*So the session becomes easier when you start by telling the index client the importance of eliciting the contacts because if you don’t prepare the index client, it becomes difficult to convince the client.*” They did not introduce the session, obtain consent or discuss confidentiality as the initial step.

The participants in Enhanced arm also completed this step, but this explanation only occurred after the introductory section. The next part consisted of explaining the purpose of index case testing and who would benefit from this service. HCWs mentioned their awareness improved on which contact clients to elicit from index clients: “*we have learned about other groups of contacts that we are supposed to follow.*” They reported that using the counseling checklist helped them to make index clients provide a fuller range of contact clients.

A third part was working with the index clients to understand the referral options. Participants also mentioned gaining an improved understanding of the different referral methods and being able to support the client based on the method selected “*At first we were just offering these methods but now we understand the ICT methods. We understand now that there are different ICT methods that we can offer an index client to get to their contacts.*” They viewed this improved understanding as contributing to eliciting and tracing more contact clients. Because they have a good understanding of the referral methods, they spend enough time with the index clients describing approaches in detail to the clients. One HCW mentioned, “*And now we are able to elicit and trace more contacts because of the time we spend discussing the ICT methods with the client*”. HCWs also mentioned being able to support the client with the referral methods selected and discuss the appropriateness or practicality of the method in detail with the client.

The last part of the index client protocol consisted of summarizing a plan to recruit contact clients. HCWs in the Enhanced arm expressed that at the end of the session, they would allow the clients to ask questions to clarify any information about the referral methods and next steps: “*The client then can ask questions because the guide encourages us to let the client talk more and ask more questions. After that, we summarize the session*.” Additionally, HCWs’ enhanced understanding of ICT protocols was led to the belief that the client will follow the action plan discussed, “*I know that the client has the information and with the skill that I use by comprehending the client’s understanding, I know that the client has the understanding of it and that if s/he promises to tell her/his contacts, s/he will really tell them because she has clear information.*”

Improvements stemming from the checklist also came up for contact client counseling, though less frequently. They described learning to approach a community contact client by verifying the client’s identity and ensuring privacy before the discussion—the first step in the checklist. In contrast, they described that before the training they would talk to the client while others were present. Similarly, HCW in the Standard arm also described that they would approach community contact clients while others are present. They also described engaging others, such as community leaders or neighbors, to help them locate the contact client if they could not find the client without disclosing the reason for seeking the client. This practice was discouraged and did not come up in the Enhanced arm.

#### Theme 2: Video vignettes facilitated new counseling skills.

Video demonstrations of counselling showed how to probe, reassure, and normalize, skills that they felt were lacking previously.

HCWs in the Enhanced arm highlighted that having the video clips readily available at the facility was a key factor in enhancing their counseling skills. Participants found modelling examples helped them improve their own skills in counseling index and contact clients. With this backdrop, they suggested that the videos should be readily available so that they can watch them when they are free for refresher training and evaluate themselves after practicing what they have watched. One participant said, “*If the video clips can be provided in the facilities so that we can refer and be reminded how best we could do the sessions.*” Another HCW mentioned not being able to understand some of the didactic narration portions, but they were able to follow the role-plays.

Notably, in the Standard arm, HCWs expressed a desire to have videos to model and reinforce counseling skills. One HCW mentioned, “*If there can be tablets with videos which we can be watching to remind us on some of the skills”*. This was viewed as a better way to learn new skills and minimize the need to refer clients to skilled HCWs all the time.

Participants emphasized learning to probe to elicit more sexual partners from the videos by watching/comparing how others did it. After watching the vignettes, they asked about different types of sexual contacts, not only asking about the spouse “*For example we ask “apart from your spouse, do you have another sexual partner?” So in response to this question, we find that maybe one client is having three sexual partners*”. To this effect, HCWs believed this had helped them find more contacts.

Probing skills were used in other parts of the interaction as well. Before the Enhanced training, HCWs would ask clients a few questions, but after they would spend more time to understand the clients’ situation and explore their needs. This encouraged a freer relationship. With this change in HCW-client interaction, HCWs mentioned being able to elicit other contacts including social contacts.

In Enhanced Arm, most HCWs noted feeling able to build rapport with the clients to make them feel free. They described grasping these skills from watching the videos, and they viewed this as a way for the client to be comfortable with them. Building a good rapport was also perceived to facilitate trust and ultimately help elicit more contact clients. In addition, assuring the client of confidentiality was another factor that made the client feel free during the session. One HCW described “*we are able to build good rapport and assure the client about confidentiality and telling the client that we are talking to every client so that s/he should not be surprised. When we do that, we build good relationship and the client feels free.*”

In contrast, in the Standard arm, HCWs described rapport simply as being friendly. One HCW mentioned, “*I make sure that I should be friendly with the client so that I build good rapport’*. They viewed that if the client perceived them as a friend, it would encourage the client to open up and attend to the information the HCW provided. Another item deemed as a way build rapport with clients was self-introduction when client walk in the room. Greetings and self-introduction were standard practice of welcoming any clients at the facilities. However, the new rapport-building skills in the Enhanced arm, such as reassuring confidentiality, being client-centered and normalizing the session did not come up.

#### Theme 3: HCWs expressed greater comfort and confidence handling a range of clients

In the Enhanced arm, participants felt comfortable counseling a fuller range of clients. They expressed feeling more comfortable interviewing any index client, which was not the case before the training.

In the Standard Arm, some HCWs demonstrated limited comfort handling some clients and opted to refer a client to another provider if they could not make counseling progress “*you can refer the client to another provider but if we could have special skills, it could help.”* This practice of referring to a more experienced colleague was done when the participants perceived their skills were limited. At times, they would also refer clients to colleagues when they believed the client was not being open with them. One HCW described, “*when I feel that they are disengaging with me, I tell them that there are other people who can help them. So I refer them to other providers.*” Based on these items, they requested videos or training to learn new skills to counsel diverse types of clients

Frequently, HCWs in the Enhanced arm reported more confidence handling adolescents which they explained required more nuance. Previously, HCWs reported adolescent clients were hard to counsel because they perceived themselves as knowing everything, or feared being judged to disclose contacts, or did not want to be delayed at the facility. After the training, they expressed comfort in working with adolescents. For example, one HCW explained, “*With the adolescents, you simply need to have a good approach by taking her/him to a private place and discuss*”. Participants described their improved ability to understand the adolescents’ needs, such as wanting to be fast-tracked and respecting their privacy. They tailored the counseling session by emphasizing the benefits of partner services, such as linking treatment or prevention services.

HCWs also described being able to more comfortably approach elicited sexual contact clients because they have the skills to talk to the clients. One HCW expressed, “*It was difficult in the past because we knew where to find the clients but we did not have proper skills to approach the client*.” Some HCWs described being able to more skillfully counsel a contact client, without disclosing confidential information of the index client. They viewed practice sessions from the training as a mechanism that reinforced the lessons.

Many HCWs in the Standard arm described challenges when eliciting and probing for contact clients that they were unable to overcome. Some of the difficulties experienced included clients not taking the questions seriously or feeling that HCW wanted them to end their romantic relationship, as a result not providing contact clients information. One HCW said “*There are some clients who don’t take the counseling seriously…For example when you are asking them to elicit their contacts, they would say, ‘to the extent of telling you my sexual partners?’*” Additionally, participants also felt that some clients hide information, making it hard to elicit contacts from this group.

#### Theme 4: HCWs perceived their counselling skills were more impactful

Notably, HCWs in the Enhanced arm reported observing an increased number of elicited contact clients, attributed to improved probing skills and a client-centered approach. One participant described “*We are able to probe more and we are able to make it client-centred making the client feel more free to give us more contacts.”* Participants described having limited skills prior to the training in probing for additional sexual partners, but the training helped them probe more effectively for sexual contact clients. This was viewed as the result of how their probing questions shifted from focusing on spouses to including other sexual partners. Other HCWs attributed their enhanced way of reassuring confidentiality as mechanisms that increase the number of contacts elicited. Some HCWs viewed the feedback from the facilitators as constructive, which in turn helped them enhance their skills. In contrast, Standard arm HCWs reported having observed no changes.

Enhanced arm HCWs also noted observing positive tracing outcomes unlike in the past where they sometimes had failed to locate contact clients and provide counseling services. They viewed this as result of their comfort and improved way of collecting locator information collection. “*At least we are having an outcome after tracing a contact…This is so because there is an improvement in terms of collection of locator information.*”

#### Theme 5: HCWs expressed greater confidence identifying and solving facility-level challenges

Enhanced arm HCWs described working together in facility-level meetings to alter their facility environment in order to identify and solve challenges.

HCWs in the Enhanced arm described holding meetings to identify facility-level challenges and develop solutions. For example, at some facilities, HCWs met to discuss the importance of collecting accurate locator information for contact clients to aid with tracing activities. At other facilities, participants met to address the challenge of finding space for counseling index clients. They opted to request additional space from supervisors, including from other departments at the clinic for counseling. One HCW described, “*After the training, we sat down and discussed that we can get more positives from the ICT and that was when the idea of asking for rooms from the other departments”*. This was done to make clients comfortable during sessions, as clients value their privacy and are more likely to open up when counseling is conducted in a private space.

In contrast, in the Standard arm, HCWs referred challenges identified to the facility supervisor for them to solve. One HCW mentioned, “*We go to the Site Supervisor to provide support and the Site Supervisor makes sure s/he assists.”*

## Discussion

We conducted a qualitative explanatory analysis to understand the ways in which a digital training impacted on HCWs’ perception of delivering index case testing. We observed important differences in the Standard and Enhanced arms. In the Enhanced arm, 1) HCWs perceived the checklists as helping them operationalize ICT, 2) Video vignettes facilitated new counseling skills, 3) HCWs expressed greater comfort and confidence handling a range of clients, 4) HCWs perceived their counselling skills were more impactful, and 5) HCWs expressed greater confidence identifying and solving facility-level challenges.

Our digital training was guided by social cognitive theory. During the training development, we focused on maximizing the HCW learning processes through the interplay between multiple factors including personal, behavioral, and environmental. Firstly, the checklists were designed to assist with operationalizing ICT protocols and support the HCWs to structure the counseling sessions. Second, the videos were developed to enhance learning counselling skills. Thirdly, practicing and receiving feedback were intended to internalize and reinforce counseling skills and improve self-efficacy through enabling HCWs to be comfortable interviewing index and contact clients. Lastly, quality improvement was designed to support HCWs to meet and discuss actionable solutions for facility-level challenges. The intuitions observed in this analysis reaffirm this approach.

Our study is one of the first studies to demonstrate that a digital training approach improved HCWs’ self-efficacy, confidence and comfort in counselling index and contact clients. Several studies have reported uncertainty among HCW on how to approach PLHIV (index clients) and discuss sexual relationships or approach sexual partners (contact clients) without prior introductions to conduct index case testing counselling [7, 8, 22]. By having the skills to build rapport and support index or contact clients in a client-centered manner, HCWs were able to interview different type of clients and discuss sexual histories with ease. Our results suggest that targeted implementation strategies developed based on formative research and theory have the potential to improve HCW self-efficacy in implementing evidence-based interventions.

Gaining skills related to ICT delivery align with previous studies that demonstrated the positive impact of digital training approaches [23–25]. Our findings suggest that HCW were able to understand which sexual partners needed to be tested and knew how to help indexes decide which tracing methods to select. This may be attributed to HCWs’ improved probing skills that not focused on spouses only but included other sexual partners. This further reinforces the importance of encompassing the essential learning features in training program materials of evidence-based interventions to enhance delivery.

Our results support other published reports emphasizing the role of checklists and job-aids in improving health care service delivery [26, 27]. HCWs emphasized that having a checklist or a guide to providing index case testing was a key factor in their improvement. Through following the protocol/checklist, HCWs were able to introduce the session, assure the clients of confidentiality and explain the purpose of the session in a client-centered manner, which enabled HCWs to build good rapport with clients and made HCW-client communication successful, enabling the clients to be free to discuss sensitive topics.

Using video vignettes, we modelled concepts traditionally taught didactically. Our findings support using vignettes to train HCWs to demonstrate the skills [15, 23–25, 28]. Another advantage of videos is the ease of conducting refresher training. HCWs emphasized that having videos readily available at the facility would enable re-orientation to counseling skills as needed, which is not possible with centralized classroom training. This supports what was found in other similar studies for training HCW using digital technology in LMIC [29, 30].

In addition to improving HCW-client interactions, the training also enabled HCWs to discuss facility-level challenges and implement actionable solutions. This supports findings from other studies conducted in LMICs [31], which shows that with adequate knowledge, HCWs can lead quality improvement discussions and effect change in their practices. HCWs gained the knowledge, skills, and attitudes necessary to integrate what they learned more broadly across their facility.

Our study provides important information through direct investigation of the perspective of HCWs implementing ICT in a real-world setting. Notably, ICT perspectives were incorporated from HCWs drawn from facilities in different settings and with varying participant volumes. This provided a thorough view of the digital training’s associated impact on ICT implementation in a low-resource setting. In addition, the qualitative interviewers and analysts were well-versed in the local language and context.

The analysis has some limitations. Firstly, the majority of the interviews were conducted virtually via phone call, whereby some visual cues might have been missed. However, the interviewers were fluent in the local language and trained in robust probing skills in order to capture detailed responses. Next, we are only to obtain the perspective of ICT from HCW, not from the clients who receive the services. Further research may be needed to investigate additional perspectives from clients who receive services from the trained HCWs.

## Conclusion

The ultimate goal of training programs is to develop the skills and competencies of HCWs to deliver evidence-based interventions in healthcare settings. The digital training approach made HCWs more capable of implementing index case testing and increasing their confidence in delivering index case testing. Digital training could be a practical tool for capacity-building of HCWs in low-resource settings.

## Supplementary Material

Supplementary Files

This is a list of supplementary files associated with this preprint. Click to download.

• Supplementary.information.IDI.guide.docx

## Figures and Tables

**Table 1:** Health worker characteristics by study arm

Characteristic	Enhanced arm (n=11) Frequency (%) Median (IQR)	Standard arm (n=15) Frequency (%) Median (IQR)
Age	32 (25,41)	29 (25,34)
Sex		
Female	4 (36%)	4 (27%)
Male	7 (64%)	10 (67%)
Education		
Some secondary school	2 (18%)	1 (7%)
Completed secondary school	5 (82%)	6 (87%)
Health Care Worker cadre		
Community health worker	4 (36%)	4 (27%)
HIV diagnostic assistant	7 (64%)	10 (67%)
Years of Job Experience	5 (3,6)	4 (1,5)
District		
Balaka	4 (36%)	6 (40%)
Machinga	7 (64%)	9 (60%)
Facility type		
Dispensary	1 (9%)	3 (20%)
Health center	8 (73%)	8 (53%)
Hospital	2 (18%)	4 (27%)
Facility Location		
Peri-urban/Urban	4 (36%)	4 (27%)
Rural	7 (64%)	11 (73%)

IQR interquartile range

**Table 2:** Themes for index case testing improved self-efficacy and illustrative quotations

Theme	Quotation Enhanced arm	Quotation Standard arm
HCWs perceived the checklists as helping them operationalize ICT	*You provided us with the guide to follow and that is what we are following. So index counseling is made easy because using the checklist,"* (8017)	*"[We need] the guideline so that you can be able to follow"* (8008)
Video vignettes facilitated new counseling skills	*"I have a plea to make. If the video clips can be provided in the facilities so that we can refer and be reminded how best we could do the sessions...If you can be having challenges with a session, you can assess yourself by watching the video and evaluate yourself where you did well and where you need to improve"(8017)*	*"If there can be tablets with videos which we can be watching to remind us on some of the skills",* (8007)
	*"[in response to how the video has helped them] We are able to probe more and we are able to make it client-centred making the client feel more free to give us more contacts"* (8021)	
HCWs expressed greater comfort and confidence handling a range of clients	*"It was difficult in the past because we knew where to find the clients [sexual contact] but we did not have proper skills to approach the client."* (8021)	*"there are times whereby you have tried to assure the client but you see that the client seems not to feel free to give you the required information. Of course you can refer the client to another provider" (8007)*
	*"Previously, we were not screening them [adolescents] if they have contacts because we were thinking that they are young, they are at school but now [after the digital training] we are able to ask them and some of them give us contacts."*(8009)	*"[Which clients are "difficult" to counsel]* *For the teenaged group, they are fond of hiding information and for you to reach a point that you have convinced them to elicit contacts, it is not a joke" (8015)*
HCWs perceived their counselling skills were more impactful	*"Previously we would just say we will come or your contact should come at the hospital. And now we are able to elicit and trace more contacts because of the time we spend discussing the ICT methods with the client. We could not do this previously." (8009)*	" *They are the same things we have been doing. [Is there anything that has changed?]. No."* (8008)
HCWs expressed greater confidence identifying and solving facility-level challenges	*"The issues of space were there before the training but after the training they are no longer there. After the training, we sat down and discussed that we can get more positives from the ICT and that was when the idea of asking for rooms from the other departments. Those in high positions intervened for the change to happen."* (8021)	" *We go to the Site Supervisor to provide support and the Site Supervisor makes sure s/he assists so that the work should be of high quality* (8006)

## Data Availability

The data that support the findings of this study are available from the corresponding author upon reasonable request.

## Abbreviations

HCW: Health Care Worker
HIV: Human Immunodeficiency Virus
ICT: Index Case Testing
IDI: In-Depth Interview
IRB: Institutional Review Board
PLHIV: People Living With HIV
UNAIDS: Joint United Nations Program on HIV/AIDS
